# Cost of septic and aseptic revision total knee arthroplasty: a systematic review

**DOI:** 10.1186/s12891-021-04597-8

**Published:** 2021-08-18

**Authors:** Charles Okafor, Brent Hodgkinson, Son Nghiem, Christopher Vertullo, Joshua Byrnes

**Affiliations:** 1 Centre for Applied Health Economics, School of Medicine and Dentistry, Griffith University, 170 Kessels Road, Nathan, Queensland 4111 Australia; 2 Menzies Health Institute, Griffith University, Gold Coast, Queensland Australia; 3 Knee Research Australia, Gold Coast, Queensland Australia

**Keywords:** Revision, Total knee arthroplasty, Cost, Economic burden, Knee, Systematic review

## Abstract

**Background:**

The increasing incidence of primary total knee arthroplasty (TKA) has led to an increase in both the incidence and the cost burden of revision TKA procedures. This study aimed to review the literature on the cost of revision TKA for septic and aseptic causes and to identify the major cost components contributing to the cost burden.

**Methods:**

We searched MEDLINE (OvidSp), Embase, Web of Science, Cochrane Library, EconLit, and Google Scholar to identify relevant studies. Selection, data extraction and assessment of the risk of bias and cost transparency within the studies were conducted by two independent reviewers, after which the cost data were analysed narratively for 1- or 2-stage septic revision without re-revision; 2-stage septic revision with re-revision; and aseptic revision with and without re-revision, respectively. The major cost components identified in the respective studies were also reported.

**Results:**

The direct medical cost from the healthcare provider perspective for high-income countries for 2-stage septic revision with re-revision ranged from US$66,629 to US$81,938, which can be about 2.5 times the cost of 1- or 2-stage septic revision without re-revision, (range: US$24,027 – US$38,109), which can be about double the cost of aseptic revision without re-revision (range: US$13,910 – US$29,213). The major cost components were the perioperative cost (33%), prosthesis cost (28%), and hospital ward stay cost (22%).

**Conclusions:**

Septic TKA revision with re-revision for periprosthetic joint infection (PJI) increases the cost burden of revision TKA by 4 times when compared to aseptic single-stage revision and by 2.5 times when compared to septic TKA revision that does not undergo re-revision. Cost reductions can be achieved by reducing the number of primary TKA that develop PJI, avoidance of re-revisions for PJI, and reduction in the length of stay after revision.

**Trial registration:**

PROSPERO; CRD42020171988.

**Supplementary Information:**

The online version contains supplementary material available at 10.1186/s12891-021-04597-8.

## Background

The increasing incidence of primary total knee arthroplasty (TKA) has resulted in a corresponding increase in the incidence of revision TKA procedures [1, 2] for both septic and aseptic reasons (such as instability, pain, stiffness, fracture and loosening-lysis). This has increased the financial burden to patients, healthcare payers, and healthcare providers (HCP), especially when the revision procedures are often not anticipated, or budgeted for. Revisions for aseptic reasons are usually single stage, while revisions for sepsis or periprosthetic joint infection (PJI) can be planned as a single stage or double stage with a risk for further revision if the infection is not resolved. The United Kingdom revision knee working group (RKWG) has described the main reasons for revision TKA using the ‘SPECIFIC’ acronym, which include stiffness and soft tissue problems, patella and malposition/ or malrotation, extensor mechanism dysfunction, component loosening, infection, fracture, instability, and component wear or breakage [3]. Early revision procedures within 2 years of the primary TKA are typically for infection, instability, pain or stiffness, with infection being the primary cause of revision of modern prostheses [4]. Late revisions are more typically due to loosening, lysis, or fracture [5, 6]. To reduce the lifetime cost of arthroplasty, it is imperative that the revision rate be reduced as much as possible.

A fundamental mechanism for cost control and fiscal planning for revision TKA is to estimate the costs of the different septic and aseptic revision TKA procedures. Knowledge of the costs across countries and settings, and the major cost components will assist to implement measures to minimize future revision burden.

This study, therefore, aimed to review the existing literature that estimated the cost of revision TKA and identify the major cost components that contribute to the total cost burden. The problem-intervention-comparator-outcome (PICO) framework was used to formulate the following research questions:

1. What is the cost of revision TKA for PJI and aseptic causes?
2. Which cost components are major contributors to the total cost of revision TKA?

## Methods

### Protocol and registration

A study protocol for this systematic review can be accessed at 10.1007/s41669-020-00242-7. The design of this systematic review followed the recommendation in the Preferred Reporting Items for Systematic Review and Meta-Analysis (PRISMA) 2009 statement [7]. Details of the PRISMA checklist are provided (see Additional file 1). The systematic review was registered in the International Prospective Register of Systematic Reviews (PROSPERO; CRD42020171988).

### Inclusion and exclusion criteria

Studies included in the review met the following criteria:

1. Original research on economic evaluations of data on revision TKA.
2. The studies presented cost data.
3. Costs were from either a patient, payer, or healthcare provider perspective.
4. Full-text articles.
5. Studies on humans and presented in the English language.

Studies were excluded for the following reasons:

1. Studies outside the scope of revision TKA.
2. Costs not specific for revision TKA or TKA costs without revision cost or cost of reoperation without revision.
3. Studies with unclear methodology. Studies with unclear methodology refer to studies with no defined approach, perspective, data items or analytical procedure.

### Information sources

We searched MEDLINE (OvidSp), Embase, Web of Science (WoS), Cochrane Library, EconLit, and Google Scholar to identify relevant studies.

### Search strategy

Using relevant Medical Subject Headings (MeSH) and text words, we created search terms. Similar search terms were combined to form union clusters. The different union clusters were combined to form an intersection. Details of the search strategy were described in the study protocol [8]. The MEDLINE search strategy was adapted for search in other databases.

MEDLINE (OvidSp), Embase, and WoS were searched on 2 November 2020, while the other databases were searched on 3 November 2020. Auto-alert systems were set-up for MEDLINE (OvidSp), Embase, and WoS by two authors, CO and BH. The other three databases were searched again by CO and BH on 4 and 5 January 2021. Two additional relevant studies were found from the auto-alert systems [9, 10]. The auto-alert systems were stopped on 10 January 2021.

### Selection process

Results of the search from the different databases were exported into a single EndNote library. The EndNote was used to de-duplicate the studies. After de-duplication, we initiated an auto-search for full-text of the articles. The selection was done independently by CO and BH against the inclusion/exclusion criteria. The selection was done in two phases. First, CO and BH screened titles and abstracts of the studies for originality, relation to revision TKA and economic evaluation or presentation of cost data. Next, we assessed the full text of potential articles for clarity of method, study perspective and cost involving revision TKA. JB and SN reviewed the selection by CO and BH. Relevant studies which were excluded in the cost synthesis but met the inclusion criteria were listed in a table describing the characteristics of the excluded studies. Details of the selection process were described in the study protocol [8].

### Data collection process

We piloted an electronic data extraction form to collect data from the selected studies. CO and BH independently extracted and managed the data from the included studies. Disagreements on some extracted results were resolved by JB. The data were collected based on the International Society for Pharmacoeconomics and Outcome Research (ISPOR) Consolidated Health Economic Evaluation Reporting Standards (CHEERS) guideline [11]. The Larg and Moss guideline for cost of illness (COI) studies and the Campbell and Cochrane Economics Methods Group (CCEMG) guideline were also employed in our data extraction process [12, 13].

### Data items, outcome, and prioritization

Data were collected for the following types of revision TKA:

i. One-stage revision without re-revision: For aseptic revision, this includes one component, two components, all components exchange or secondary patella resurfacing. In the case of sepsis, it involves open debridement of the infected TKA followed by immediate revision by removal and or reimplantation of all components (one-stage revision for PJI) or just the exchangeable polyethylene component (DAIR – Debridement, Antibiotics, and Implant Retention). One-stage revision of all components or DAIR are more commonly used in patients without systemic sepsis, extensive comorbidities or immunocompromise, infection with resistant organism, culture-negative infection, and poor soft tissue coverage [14]. To perform a DAIR, the femoral and tibial components need to be well-fixed and preferably undertaken acutely, prior to bio-film formation on the components.
ii. One-stage revision with re-revision: In this case there is a surgical failure of the one-stage revision TKA or another SPECIFIC diagnosis [3] requiring revision, which leads to a subsequent revision. In re-revision for aseptic failure, the original revision may not have addressed the cause of failure adequately, such as component malposition if only one component was exchanged. In re-revision for recurrent PJI, the initial one-stage procedure has failed to eradicate the PJI.
iii. Two-stage revision without re-revision: This procedure is the most common procedure for the treatment of chronic knee PJI. The first stage consists of the removal of the infected implant, surgical debridement, and insertion of a temporary antibiotic spacer device. After a delayed time, usually 3–6 months later, the second stage (reimplantation) is performed when the treating medical team confirms that the infection has resolved [14]. The second stage involves the removal of the antibiotic spacer and the application of another prosthetic implant [15].
iv. Two-stage revision with re-revision: In this case there is a surgical failure of the two-stage revision TKA for PJI, or another SPECIFIC diagnosis [3] requiring revision, which leads to a subsequent revision. This is more likely in multi-organism PJI, PJI with resistant microorganisms, or in immunocompromised patients.

Data was also collected on the factors responsible for revision and the cost drivers for revision TKA.

Data were extracted based on the following:

- Publication: title, authors, year, and country the study was conducted.
- Study design: cohort study, case-control study, and cross-sectional study.
- Aim of the study, sample size, gender, study perspective, data source, the time horizon of observation, timeframe of cost estimation, number of revisions, length of hospital stay, comparators, and type of economic evaluation.
- Cost measure: direct medical costs which include medical costs involved in the direct provision of healthcare.

### Risk of bias and cost transparency within the studies

We performed the risk of bias and cost transparency assessment of the individual studies at the outcome level using the Consensus Health Economic Criteria (CHEC) and the Larg and Moss checklists [12, 16] and the Fukuda and Immanaka criteria [17]. Studies were classified as ‘low risk’ (0–10%), ‘low-moderate risk’ (11–20%), ‘high-moderate risk’ (21–30%), or ‘high risk’ (> 30%) based on applicable items for each study [8]. Furthermore, studies were classified as ‘excellent’ (Aα), ‘very good’ (Aβ; Bα; Bβ), ‘good’ (Bγ; Cα; Cβ; Cγ), ‘fair’ (Bδ; Cδ; Dα; Dβ; Dγ) and ‘poor’ (Dδ; Dε) in cost transparency [8]. Studies with a high risk of bias (> 30%) and or poor cost transparency (Dδ and Dε) were excluded from the data synthesis. The assessments were completed in duplicate by CO and BH. Differences were resolved with SN.

### Summary measures

The primary measure was the mean cost of revision TKA, while the second measure (where possible) was the cost difference between the primary TKA and the revision TKA.

### Data synthesis

Due to the heterogeneity in the cost estimates of the included studies, we performed a narrative synthesis of answers to our research questions. To achieve this, we used the direct medical cost of studies from the HCP perspective. Costs of revision TKA from the included studies were grouped into aseptic revision TKA and septic revision TKA. For septic revision TKA, we classified cost estimates as 1- or 2-stage septic revisions without re-revision (i.e., one revision only) and 1- or 2-stage septic revision with one re-revision (i.e., two revisions). All costs were adjusted to 2019 USD values. The cost adjustment followed the guidelines of the CCEGM and the Evidence for Policy and Practice Information and Coordinating Centre (EPPI-Centre) [18].

## Results

### Study selection

**Fig. 1:**
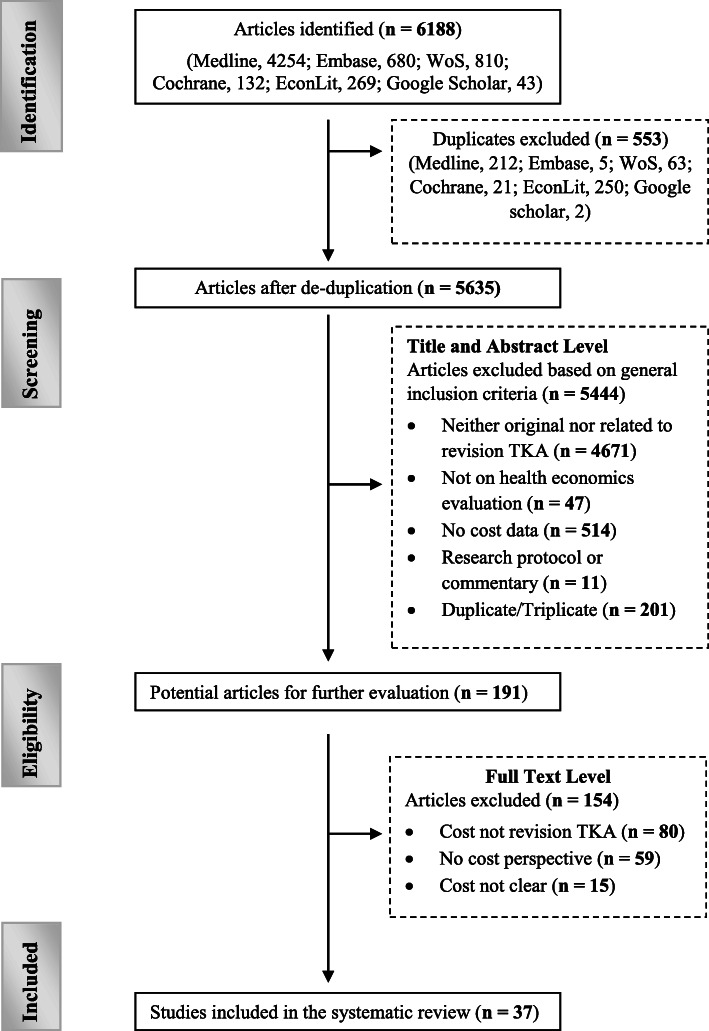
PRISMA flowchart of the study selection process

A total of 6188 studies were identified from the six databases at the end of the search. De-duplication was done, and 5635 studies were available for screening. Studies excluded at the screening phase were moved to exclusion folders based on the reason for exclusion in a hierarchy order already defined in the study protocol [8]. We found further duplicates at this stage which were also excluded. At the end of the screening, 191 potential studies were available for eligibility check. A total of 37 studies met the criteria for inclusion. See Fig. 1 for the detailed flow diagram of the selection process.

### Studies characteristics

Data were extracted from each study based on the data items described in the study protocol [8]. Data extraction showed that out of the 37 studies, 22 studies (59%) were conducted in the United States [9, 19–39]. Studies were also conducted in Turkey [40], Canada [41], Brazil [42], Italy [43], New Zealand [44], Germany [45–47], Czech Republic [48, 49], United Kingdom [50], Republic of Ireland [51], Finland [52], Portugal [53] and Pakistan [10]. The sample size for each study used in this review was the interest group (revision TKA) sample size as some studies assessed primary TKA and hip joint replacements in addition to revision TKA. The non-interest group sample size and general sample size details can be found in ‘Additional file 2’. The sample size ranged from a minimum of 3 patients in Gow et al. [44] to a maximum of 301,718 patients in Kamath et al. [25]. The patients′ demographic characteristics (specifically age and sex) were similar across the studies. Patients’ mean age ranges from 61.3 years in Iqbal et al. [10] to 74.6 years in Li et al. [31]. In 84% of the included studies, females were the majority who underwent revision TKA [29, 30, 35, 42, 46, 53]. Infection was the major cause of revision in 70% of the studies [10, 20, 26, 38, 43, 45, 46, 51, 53], while mechanical loosening, instability, fracture, pain and other aseptic causes represent 30% [30, 35, 50]. A 2-stage septic revision was the most widely used surgical approach in most cases of septic revision. Most of the studies (82%) estimated cost from the HCP perspective [10, 20, 25, 34, 38, 40, 42, 43, 45, 51, 53], while the remainders (18%) were from the payer and patient perspective [31, 33, 36, 39, 41, 48, 50]. All studies were COI studies except for Burns et al., which was a cost-effectiveness analysis [41]. All of the included studies reported direct medical costs of management only. The time horizon of observation ranged from 12 months [20, 35] to 180 months [27]. The length of hospital stay varied from 5 days [21, 24, 53] to 48 days [45]. Prosthetic joint infection was the main reason for longer stay [19, 50, 51]. The timeframe of the cost estimation was similar to the length of hospital stay for most studies except for four studies which had a short-term follow-up cost (post hospital discharge cost) of about one week to four months [21, 26, 32, 43]. The studies’ designs, methodologies and cost evaluation methods were assessed for risk of bias and transparency, and each study was scored accordingly. See Table 1 for details of the studies’ characteristics, and Additional file 2 for further details.

**Table 1:** Characteristics of included studies

Author, year & reference	Country	Interest –case sample size	Age (CI) [SD]	Sex proportion	Cause of revision	Study perspective	Time horizon of observation (months)	Type of Economic Evaluation	Comparator	Study design	Cost Component
Adeyemi et al., 2019 [20]	United States	1140	64.8	Male, 42.1%; Female, 57.9%	Infection	HCP	12	COI	Non-SSI. RTHA	Cohort	Direct cost
Alp et al., 2016 [40]	Turkey	11	65	Male, 29.4%; Female, 70.6%	Infection	HCP	24	COI	Non-SSI	Cross sectional	Direct cost
Bosco III et al., 2014 [21]	United States	118	N/A	N/A	N/A	HCP. payer	48	COI	THA; TKA; RTHA	Cohort	Direct cost
Bozic et al., 2010 [22]	United States	60,355	65.8	Male, 42.6%; Female, 57.4%	Infection; mechanical loosening; implant fracture	HCP	15	COI	N/A	Cross sectional	Direct cost
Burns et al., 2006 [41]	Canada	73	N/A	N/A	N/A	Payer	24	CEA	PTKA	Cohort	Direct cost
Clair et al., 2016 [23]	United States	24	65	Male, 29.2%; Female, 70.8%	N/A	HCP	12	COI	RTHA	Cross sectional	Direct cost
Dal-Paz et al., 2010 [42]	Brazil	34	67.1 ± 12.0	Male, 32.4%; Female, 67.6%	Infection	HCP	24	COI	N/A	Cross sectional	Direct cost
Efremov et al., 2019 [43]	Italy	30	71.2 ± 6.3	Male, 53%; Female, 47%	Infection	HCP	30.4	COI	RTHA	Cohort	Direct cost
Gow et al., 2016 [44]	New Zealand	3	69 (49–78)	Male, 72.2%; Female, 27.8%	Infection	HCP	12	COI	Non-SSI	Case-control	Direct cost
Haenle et al., 2012 [45]	Germany	28	71.7	Male, 28.6%; Female, 71.4%	Infection	HCP	48	COI	PTKA	Case-control	Direct cost
Herbert et al., 1996 [19]	United States	20	65	N/A	Aseptic; Infection	HCP	36	COI	PTKA	Cross sectional	Direct cost
Holinka et al., 2018 [48]	Czech Republic	20	68.5 (55–82)	N/A	Aseptic; Infection	Payer	N/A	COI	PTKA	Cross sectional	Direct cost
Iorio et al., 1999 [24]	United States	32	N/A	N/A	N/A	HCP	24	COI	PTKA	Cross sectional	Direct cost
Kallala et al., 2015 [50]	United Kingdom	168	65.6	Male, 46.43%; Female, 53.57%	Aseptic; Infection	Patient	84	COI	Aseptic revision	Cross sectional	Direct cost
Kamath et al., 2015 [25]	United States	301,718	65.7 (65–69)	Male, 50.1%; Female, 49.9%	Infection	HCP	64	COI	RTHA	Cross sectional	Direct cost
Kapadia et al., 2014 [26]	United States	21	N/A	N/A	Infection	HCP	60	COI	PTKA	Case-control	Direct cost
Kasch et al., 2017 [46]	Germany	106	66.8 [9.5]	Male, 35.8%; Female, 64.2%	Aseptic; Infection	HCP	39	COI	Aseptic revision	Cross sectional	Direct cost
Kurtz et al., 2008 [27]	United States	N/A	69.5	N/A	Infection	HCP	180	COI	RTHA; PTKA; PTHA	Cross sectional	Direct cost
Kurtz et al., 2012 [28]	United States	105,068	64.5	N/A	Infection	HCP	108	COI	RTHA	Cross sectional	Direct cost
Lavernia et al., 1995 [29]	United States	24	68.0	Male, 29.2%; Female, 70.8%	N/A	HCP	48	COI	PTHA; PTKA; RTHA	Cross sectional	Direct cost
Lavernia et al., 2006 [30]	United States	100	63.5	Male, 28.6%; Female, 71.4%	Aseptic loosening; Infection	HCP	108	COI	N/A	Cross sectional	Direct cost
Li Y. et al., 2013 [31]	United States	18,677	74.6 [6.5]	Male, 38.8%; Female, 61.2%	N/A	Payer	12	COI	PTKA	Cross sectional	Direct cost
Musil et al., 2019 [49]	Czech Republic	24		Male, 45.8%; Female, 54.2%	Infection	HCP	96	COI	N/A	Cross sectional	Direct cost
Nichols et al., 2016a [32]	United States	25,354	63.3 [10.5]	Male, 42.3%; Female, 57.7%	N/A	Payer + patient	60	COI	PTKA; PTHA; RTHA	Cross sectional	Direct cost
Nichols et al., 2016b [33]	United States	32,494	65.26 [10.98]	Male, 40.5%; Female, 59.5%	N/A	Payer	78	COI	PUTKA; SBTKA	Cross sectional	Direct cost
Oduwole et al., 2010 [51]	Republic of Ireland	179	71.5	Male, 35%; Female, 65%	Infection; Aseptic	HCP	120	COI	Aseptic revision	Cross sectional	Direct cost
Parvizi et al., 2010 [34]	United States	216	62.4	Male, 48.8%; Female, 51.2%	Infection	HCP	132	COI	Methicillin sensitive cases	Cross sectional	Direct cost
Puhto et al., 2019 [52]	Finland	26	69 (38–91)	Male, 49.5%; Female, 50.5%	Infection; Aseptic	HCP	36	COI	Primary TKA	Cohort	Direct cost
Reeves et al., 2018 [35]	United States	46,836	69.1	Male, 31.1%; Female, 68.9%	Prosthetic fracture; non-fracture cause	HCP	12	COI	PPFX; ORIF; PTKA	Cohort	Direct cost
Ritter et al., 1996 [36]	United States	26	N/A	N/A	N/A	Payer	N/A	COI	PTKA; PTHA; RTHA	Cross sectional	Direct cost
Sculco, 1995 [37]	United States	N/A	N/A	N/A	N/A	HCP	N/A	COI	N/A	Cross sectional	Direct cost
Sousa et al., 2018 [53]	Portugal	29	68.3 (39–80)	Male, 32.2%; Female, 67.7%	Infection	HCP	24	COI	PTKA; PTHA; RTHA	Case-control	Direct cost
Waddell et al., 2016 [38]	United States	70	N/A	N/A	Infection	HCP	56	COI	RTHA	Cross sectional	Direct cost
Weber et al., 2018 [47]	Germany	68	67.9 [9.2]	Male, 38.2%; Female, 61.8%	N/A	HCP	60	COI	PTHA; PTKA; RTHA	Case-control	Direct cost
Yi et al., 2015 [39]	United States	79	≥ 65	Male, 43%; Female, 57%	Infection	Payer	96	COI	PTHA; PTKA; RTHA	Cohort	Direct cost
Yao et al., 2020 [9]	United States	266	65 (54–76)	Male, 61%; Female, 39%	Infection; Aseptic	HCP + Payer	84	COI	Non-SSI	Cross sectional	Direct cost
Iqbal et al., 2020 [10]	Pakistan	32	61.3 (55.4–67.2)	N/A	Infection	HCP	60	COI	PTKA	Case-control	Direct cost

*N/A* Not available, *SD* Standard deviation, *HCP* Healthcare provider, *COI* Cost of illness, *CEA* Cost-effectiveness analysis, *SSI* Surgical site infection, *TKA* Total knee arthroplasty, *THA* Total hip arthroplasty, *RTHA* Revision total hip arthroplasty, *PTKA* Primary total knee arthroplasty, *PTHA* Primary total hip arthroplasty, *PUTKA* Primary unilateral total knee arthroplasty, *SBTKA* Simultaneous bilateral total knee arthroplasty, *TJA* Total joint arthroplasty, *PPFX* Periprosthetic fracture, *ORIF* Open reduction internal fixation

### Risk of bias and cost transparency of the included studies

**Fig. 2:**
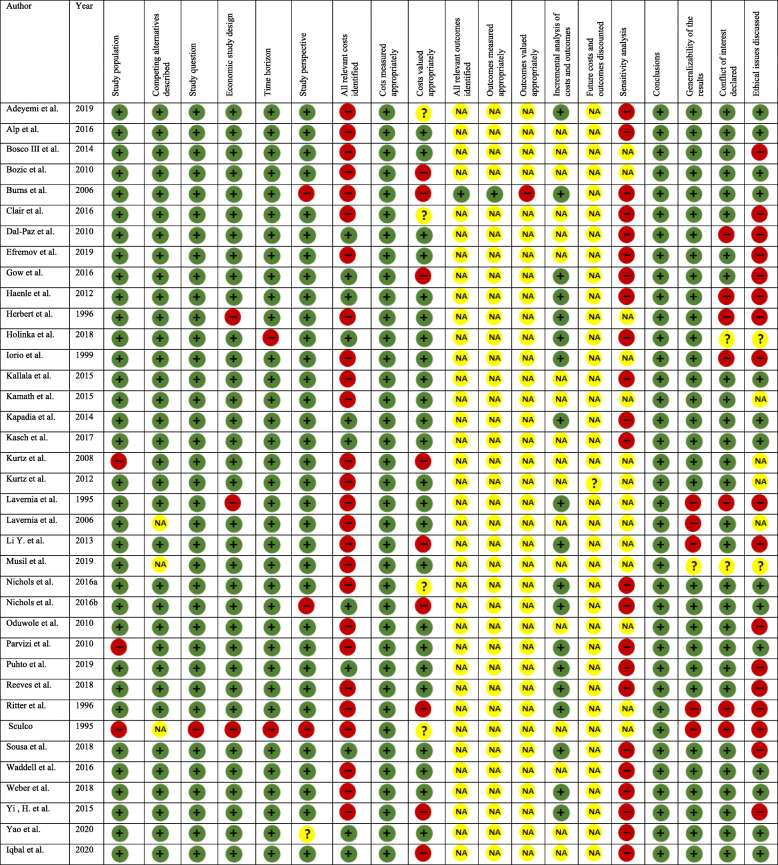
Risk of bias assessment of the included studies using the Consensus Health Economic Criteria

From the risk of bias assessment, four studies [23, 36, 37, 41] had a high risk of bias, while the cost transparency test showed that three studies had poor transparency [22, 27, 37]. These six studies were excluded from the outcome analysis. Six studies presented cost as reimbursement and were also excluded in the outcome analysis [31, 33, 36, 39, 41, 48]. Three studies [40, 44, 52] combined the cost of knee and hip revision and were also excluded in the analysis since the costs for revision TKA alone were not presented. Four more studies were also excluded because the costs were a combination of reoperation and revision [20, 42], rebate cost [46], and hybrid cost [9]. In all, 17 studies were excluded from the data synthesis. Figure 2 presents the risk of bias assessment using the CHEC checklist, while the Larg and Moss assessment was presented in Additional file 2. Additional file 3 presents the scores for the risk of bias and transparency assessment, respectively.

Studies that presented cost as charges (without presenting the cost-charge ratio) [26, 29] but passed the risk of bias and cost transparency tests were converted to cost using a cost-charge ratio [54]. All of these studies [26, 29] were United States-based, and as such the United States national-average cost-charge ratio of 0.5, based on the healthcare cost and utilization project (HCUP) estimate, was used [54].

### Cost of revision total knee replacement of the individual studies

After the risk of bias and transparency assessment, 20 studies were included in the data synthesis. 14 out of the 20 studies reported cost data for septic revision [10, 19, 25, 26, 28, 30, 34, 38, 43, 45, 49–51, 53], while 7 studies reported cost data for aseptic revision [19, 25, 30, 35, 50, 51, 53]. Six studies reported costs for revision TKA without specifying the cause and type of revision [21, 24, 29, 32, 42, 47]. Cost data were mostly from developed countries except for two studies from Brazil [42] and Pakistan [10].

The cost of revision TKA ranged from US$7837 in Weber et al. [47] to US$81,938 in Hebert et al. [19]. Revision TKA was about two times the cost of primary TKA as reported in several studies [19, 35, 47, 53]. Also, the cost of revision TKA due to PJI was higher than aseptic revision by about 2-fold, but this is dependent on certain factors which include the number of revisions, length of hospital stays and cost duration. The direct medical cost from the healthcare provider perspective for high-income countries for 2-stage septic revision with one re-revision ranged from US$66,629 [26] to US$81,938 [19], while for 1- or 2-stage septic revision without re-revision, the cost ranged from US$24,027 [53] to US$38,109 [49]. For aseptic revision with one re-revision, the cost ranged from US$35,926 to US$37,791 [30], while for aseptic revision without re-revision, the cost ranged from US$13,910 [53] to US$29,213 [19]. Table 2 presents the narrative costs of revision TKA across different studies and settings while Additional file 3 presents the excluded studies from the data synthesis.

**Table 2:** Costs of revision total knee replacement

Author & Year	Cost year of study & currency	Cost estimate (CI), [SD], 2019 USD	Cost difference between PTKA & RTKA	Number of revisions	Length of hospital stay (days)	Duration of cost estimate (days)
*One- or two-stage septic revision*						
Efremov et al., 2019	2017, Euro	2-stage: 31,020 (18,668 - 52,916)	N/A	1	26 (15–52)	147
Haenle et al., 2012	2011, Euro	37,047	26,917	1	48.2	48.2
Kallala et al., 2015	2012, Pounds	48,428 [7284] ^a^	N/A	1	21.5	21.5
Kamath et al., 2015	2010, USD	30,032	N/A	1	7.5	7.5
Kurtz et al., 2012	2010, USD	28,288 (26,651 - 29,924)	N/A	1	7.2 (6.9–7.9)	7.6
Musil et al., 2019	2018, CZK	2-stage: 38,109	N/A	1	10	10
Oduwole et al., 2010	2006, Euro	2-stage: 29,314 (15,448 - 42,936)	N/A	1	39	39
Sousa et al., 2018	2015, Euro	2-stage: 24,027	8487	1	14.3	14.3
Iqbal et al., 2020	2019, PKR	2-stage: 12,277 (10,114 – 14,440)		1	11	11
Waddell et al., 2016	2013, USD	2-stage: 37,792 (30,293 – 48,319)	N/A	1	9	9
Herbert et al., 1996	1993, USD	2-stage: 81,938	35,129	2	32.1	32.1
Kapadia et al. 2014	2011, USD	2-stage: 66,629 (25,428 – 154,526)	50,456	2	23.7 (4–49)	30.2
Lavernia et al., 2006	2005, USD	2-stage: 75,462 (72,390 – 78,535)	N/A	2	16	16
Parvizi et al., 2010	2009, USD	2-stage: 77,420 (58,065 – 96,775)	N/A	2	28.5	28.5
*Aseptic revision*						
Herbert et al., 1996	1993, USD	29,213		1	12.8	12.8
Kallala et al., 2015	2012, Pounds	15,980 [968] ^a^	N/A	1	9.6	9.6
Kamath et al., 2015	2010, USD	22,860	N/A	1	7.5	7.5
Oduwole et al., 2010	2006, Euro	19,245 (7403 – 31,424)	N/A	1	16	16
Reeves et al., 2018	2013, USD	16,806 (12,605 – 21,008)	4314	1	6	6
Sousa et al., 2018	2015, Euro	13,910	N/A	1	5	5
Lavernia et al., 2006	2005, USD	35,926 (34,061 – 37,791)	N/A	2	6.6	6.6
*Unspecified cause and type of revision*						
Bosco III et al., 2014	2012, USD	67,210	30,616	2	5	30
Lavernia et al., 1995	1991, USD	18,674 (17,180 – 20,168)	4528	1	17	17
Iorio et al., 1999	1995, USD	18,612	2321	1	5.1	5.1
Nichols et al., 2016a	2013, USD	75,766	18,564	2	5.6	90
Weber et al., 2018	2016, USD	7837 [2278]	3339	1	13.1	13.1

*CI* Confidence interval, *PTKA* Primary total knee arthroplasty, *RTKA* Revision total knee arthroplasty, *CZK* Czech koruna, *PKR* Pakistan rupee, *N/A* Not applicable.

^a^Patient cost perspective

### Major cost components of revision total knee replacement

The results from the included studies showed that the major cost components include perioperative cost (operating room cost, anaesthesia, and procedure cost), 33% (6–50%); prosthesis cost, 28% (10–45%); and cost of hospital ward stay, 22% (5–35%). The major cost components described by the included studies are presented in Table 3.

**Table 3:** Major cost drivers from the included studies

Author, year	Cost drivers in descending order for each study (proportion of total cost)
Dal-paz et al., 2010	Medications’ cost (27%);
	Hospital ward stay cost (25%);
	Laboratory tests cost (20%);
	Prosthesis cost (9.5%)
Haenle et al., 2012	Hospital ward stay cost (27%);
	Prosthesis cost (23%)
Kamath et al., 2015	Hospital ward stay cost (N/A)
Kapadia et al., 2014	Operating room cost (56%);
	Hospital ward stay cost (22%)
Kasch et al., 2017	Prosthesis (34%);
	Hospital ward stay (21%);
	Personnel cost (13%)
Lavernia et al., 1995	Prosthesis cost (45%)
Nichols et al., 2016a	Medical and surgical supplies cost (N/A)
Nichols et al., 2016a	Medical and surgical supplies cost (59%);
	Operating room cost (19%);
	Room and board cost (14%)
Oduwole et al., 2010	Hospital ward stay cost (N/A);
	Prosthesis cost (N/A)
Puhto et al., 2019	Operating/procedure /prosthesis cost (51%);
	Hospital ward stay cost (28%)
Sousa et al., 2018	Prosthesis and clinical materials cost (60%);
	Personnel cost (14%);
	Hospital ward stay cost (8%)
Waddell et al., 2016	Prosthesis cost (N/A);
	Perioperative cost (N/A);
	Hospital ward stay cost (N/A)
Weber et al., 2018	Prosthesis cost (41%);
	Hospital ward stay cost (35%);
	Perioperative cost (24%)
Yao et al., 2020	Operating room and anaesthesia cost (39%);
	Room and board cost (24%);
	Prosthesis cost (17%)
Iqbal et al., 2020	Prosthesis & clinical materials cost (77%);
	Operating room cost (6%);
	Hospital ward stay cost (5%)
**Summary cost major cost components (range)**	**Perioperative cost 33% (6–50%);**
	**Prosthesis cost 28% (10–45%)**
	**Hospital ward stay cost 22% (5–35%)**

***N/A*Proportion not available or reported**

## Discussion

This systematic review assessed the cost of septic and aseptic revision TKA to provide an overview of its financial burden and explored some of the cost components that drive the total cost, revealing that the cost of septic revision TKA can be about twice as expensive as aseptic revision TKA, an important consideration given that infection is now the most common reason for revision with modern prostheses [4]. The major cost components identified were the perioperative cost, prosthesis cost, and hospital ward stay cost. It is feasible to reduce the high-cost burden of revision TKA through the major factors and cost drivers identified from this review. At a fundamental level, the number of revisions per patient, hence the cost burden of revision, can be substantially reduced through optimal patient, prosthesis and procedure selection for the primary TKA [55, 56] and by reducing the number of primary TKA by effective non-operative knee osteoarthritis management, such as obesity reduction and exercise programs.

At a more tertiary level, cost-containment can minimize the high cost burden associated with revision TKA [24]. Implant cost reduction programs such as price reductions from the manufacturer, competitive bidding processes by the hospital, and implant standardization can help reduce implant cost [24]. The cost of hospital ward stay length after revision TKA can be minimized through enhanced perioperative procedures, and post-operative practices [55] including outpatient intravenous antibiotic services especially for patients whose residential addresses are close to the hospital.

Furthermore, with PJI it remains unclear when to perform a DAIR, a single stage revision of all components or a two-stage revision. Further research and decision-making tools are required to enable surgeons to better predict optimal candidates with PJI for the three different revision options. Unfortunately, PJI patients with a failed DAIR or a failed single stage revision then require a two-stage revision, a large burden to both the individual and society. The risk of re-revision after single- or two-stage PJI revision can also be minimized by optimizing pre-operative status and post-operative status of patients to minimize immunocompromise and attenuate risk [55]. Finally, the risk of repeat revision after a failed single stage revision for aseptic failure can be reduced by avoidance of one component revision when revision of both femoral and tibial components is more likely to reduce later revision risk, such as with revision for stiffness, instability or mal-positioning.

Continuous clinical monitoring through national joint registries and infection-specific surveillance networks can benefit revision TKA cost. The Dutch PREZIES network (‘PREventie van ZIEkenhuisinfecties door Surveillance’) [57] and the French CRIOAcs (Centres de Référence des Infections Ostéoarticulaires complexes) healthcare network [58] have proven their potential to control the cost of revision TKA due to PJI, which has led to the current initiative to implement similar surveillance network in the United Kingdom to control the surgical site infection cost burden [59]. Moreover, greater research funding should be provided to arthroplasty-related PJI prevention and management [60]. Finally, health services reorganisation to provide specialised arthroplasty revision centres should be considered to also control the cost burden of revision TKA [60].

The studies included in this review have several limitations. None of the studies evaluated the indirect cost of revision TKA to the society or patient. Accordingly, some of the studies discussed this as part of their study limitations [42, 45, 50, 53]. Second, there were variations in the cost components and duration of the cost estimate used in the respective studies due to variations in different healthcare systems. This heterogeneity made it impossible to perform a meta-analysis of the cost data. Some of the studies only considered inpatient cost without outpatient care cost [10, 25, 30, 45, 53]; some did not include follow-up cost like the cost of further readmission due to complications [50, 53]. In some of the studies from the United States whose costs were obtained from the National databases, surgeon costs were not included [19, 29, 30]. Third, some studies presented cost as a proxy using charges [19, 22, 26, 29]. Fourth, over 70% of the studies could not present the cost of each component or the unit cost of the components to show transparency [20–25, 27, 28, 30, 34, 35, 44, 50]. Future studies should consider the limitations found within this systematic review in providing robust estimates for the cost of revision TKA.

Our study has several limitations in its analyses and syntheses. First, we included studies as far as the year 1996. Clinical practice in the last decade has evolved compared to the 1990s. Limiting the studies to recent dates would have provided a more updated result. However, limiting to more recent studies would have also reduced the number of studies evaluated which, in turn, reduces the robustness of our analysis. We used an updated costing tool that incorporates purchasing power parity and inflation to reflect all costs in 2019 USD, which minimizes this limitation [18]. Second, for the United States costs data presented as charges, without presenting the cost-to-charge ratio, we used a national cost-charge ratio of 0.5 to convert charges to cost. This could underestimate or overestimate the actual cost. Third, although, most cost estimates from the included studies were the direct medical costs from the HCP perspective, which enabled comparison of costs in the different revision TKA groups, our results undermined the direct non-medical and the indirect costs of revision TKA. Fourth, due to structural differences in the healthcare systems of different countries, the use of cost as an outcome for narrative synthesis is a limitation. We also had limited ability to ensure the cost items of studies included in the narrative synthesis are precisely the same.

## Conclusion

The cost burden of septic revision TKA with re-revision can be 2.5 times greater than for septic revision and 4 times greater than aseptic revision when re-revision is not performed. Cost reductions can be achieved by reducing the number of primary TKA that develop PJI, avoidance of re-revisions for PJI, and reduction in the length of stay after revision.

## Supplementary Information

**Additional file 1.**

**Additional file 2.**

**Additional file 3.**

## Data Availability

All data generated and analyzed during this study are included in this article and its supplementary files (Additional file 2 and Additional file 3).

## Abbreviations

TKA: Total Knee Arthroplasty
PJI: Periprosthetic Joint Infection
ISPOR: International Society for Pharmacoeconomics and Outcome Research
CHEERS: Health Economic Evaluation Reporting Standards
CHEC: Consensus Health Economic Criteria
CCEMG: Campbell and Cochrane Economics Methods Group
EPPI-Centre: Evidence for Policy and Practice Information and Coordinating Centre
COI: Cost-of-illness
HCP: Healthcare Provider
HCUP: Healthcare Cost and Utilization Project
LOS: Length of Hospital Stay
RKWG: Revision Knee Working Group
