# Supplementary material for: Cost of septic and aseptic revision total knee arthroplasty: a systematic review
Source: BMC Musculoskelet Disord. 2021 Aug 18;22:706. doi: 10.1186/s12891-021-04597-8 (PMC8371784; doi:10.1186/s12891-021-04597-8)
Supplement: Supplementary file 3 — Additional file 3. [file 12891_2021_4597_MOESM3_ESM.docx]

Risk of bias checklist score for the included studies.

| Author, year | CHEC | Larg & Moss | Combined | Risk Score (%) | Inference |
| --- | --- | --- | --- | --- | --- |
| Adeyemi et al, 2019 | 12 {15} | 6 {7} | 18 {22} | 18.2 | Low-moderate risk |
| Alp et al., 2016 | 12 {14} | 6 {7} | 18 {21} | 14.3 | Low-moderate risk |
| Bosco III et al., 2014 | 11 {13} | 6 {7} | 17 {20} | 15.0 | Low-moderate risk |
| Bozic et al., 2010 | 11 {13} | 5 {7} | 16 {20} | 20.0 | Low-moderate risk |
| Burns et al., 2006 | 12 {17} | 4 {7} | 16 {24} | 33.3 | High risk |
| Clair et al., 2016 | 8 {13} | 5 {7} | 13 {20} | 35.0 | High risk |
| Dal-Paz et al., 2010 | 11 {14} | 7 {7} | 18 {21} | 14.3 | Low-moderate risk |
| Efremov et al., 2019 | 11 {14} | 6 {7} | 17 {21} | 19.0 | Low-moderate risk |
| Gow et al., 2016 | 12 {15} | 5 {7} | 17 {22} | 22.7 | High-moderate risk |
| Haenle et al., 2012 | 12 {14} | 7 {7} | 19 {21} | 9.5 | Low risk |
| Herbert et al., 1996 | 10 {14} | 6 {7} | 16 {21} | 23.8 | High-moderate risk |
| Holinka et al., 2018 | 11 {15} | 7 {7} | 18 {22} | 18.2 | Low-moderate risk |
| Iorio et al., 1999 | 11 {14} | 6 {7} | 17 {21} | 19.0 | Low-moderate risk |
| Kallala et al., 2015 | 12 {14} | 6 {7} | 18 {21} | 14.3 | Low-moderate risk |
| Kamath et al., 2015 | 11 {12} | 6 {7} | 17 {19} | 10.5 | Low-moderate risk |
| Kapadia et al. 2014 | 14 {15} | 6 {7} | 20 {22} | 9.1 | Low risk |
| Kasch et al., 2017 | 13 {14} | 7 {7} | 20 {21} | 4.8 | Low risk |
| Kurtz et al., 2008 | 9 {12} | 5 {7} | 14 {19} | 26.3 | High-moderate risk |
| Kurtz et al., 2012 | 11 {13} | 6 {7} | 17 {20} | 15.0 | Low-moderate risk |
| Lavernia et al., 1995 | 9 {14} | 6 {7} | 15 {21} | 28.6 | High-moderate risk |
| Lavernia et al., 2006 | 9 {11} | 6 {7} | 15 {18} | 16.7 | Low-moderate risk |
| Li Y. et al., 2013 | 10 {14} | 5 {7} | 15 {21} | 28.6 | High-moderate risk |
| Musil et al., 2019 | 8 {12} | 6 {6} | 14 {18} | 22.2 | High-moderate risk |
| Nichols et al., 2016a | 11 {15} | 5 {7} | 16 {22} | 27.3 | High-moderate risk |
| Nichols et al., 2016b | 12 {15} | 5 {7} | 20 {22} | 9.1 | Low risk |
| Oduwole et al., 2010 | 11 {13} | 6 {7} | 17 {20} | 15.0 | Low-moderate risk |
| Parvizi et al., 2010 | 12 {15} | 5 {7} | 17 {22} | 22.7 | High-moderate risk |
| Puhto et al., 2019 | 13 {15} | 7 {7} | 20 {22} | 9.1 | Low risk |
| Reeves et al., 2018 | 12 {15} | 6 {7} | 18 {22} | 18.2 | Low-moderate risk |
| Ritter et al., 1996 | 9 {14} | 5 {7} | 14 {21} | 33.3 | High risk |
| Sculco, 1995 | 2 {12} | 0 {4} | 2 {16} | 87.5 | High risk |
| Sousa et al., 2018 | 13 {15} | 7 {7} | 20 {22} | 9.1 | Low risk |
| Waddell et al., 2016 | 12 {14} | 6 {7} | 18 {21} | 14.3 | Low-moderate risk |
| Weber et al., 2018 | 13 {15} | 6 {7} | 19 {22} | 13.6 | Low-moderate risk |
| Yi et al., 2015 | 11 {15} | 5 {7} | 16 {22} | 27.3 | High-moderate risk |
| Yao et al., 2020 | 12 {14} | 6 {7} | 18 {21} | 14.3 | Low-moderate risk |
| Iqbal et al. 2020 | 12 {14} | 5 {7} | 17 {21} | 19.0 | Low-moderate risk |

**Note**: Adapted from CHEC and Larg & Moss checklists. The scores in curly brackets represent total applicable items for each study, maximum of 19 for CHEC and 9 for Larg & Moss checklists.

**Rating guide**: 0 – 10% = Low risk; 11 – 20% = Low-moderate risk; 21 – 30 = High-moderate risk; > 30% = High risk

Assessment of cost transparency

| Authors, year | Clarification of scope of costing | Accuracy of methodologies for evaluating cost | Combined | Rank | Inference |
| --- | --- | --- | --- | --- | --- |
| Adeyemi et al, 2019 | D | γ | Dγ | 4 | Fair |
| Alp et al., 2016 | B | α | Bα | 2 | Very good |
| Bosco III et al., 2014 | C | α | Cα | 3 | Good |
| Bozic et al., 2010 | D | δ | Dδ | 5 | Poor |
| Burns et al., 2006 | D | α | Dα | 4 | Fair |
| Clair et al., 2016 | D | α | Dα | 4 | Fair |
| Dal-Paz et al., 2010 | A | α | Aα | 1 | Excellent |
| Efremov et al., 2019 | C | α | Cα | 3 | Good |
| Gow et al., 2016 | C | α | Cα | 3 | Good |
| Haenle et al., 2012 | B | α | Bα | 2 | Very good |
| Herbert et al., 1996 | C | γ | Cγ | 3 | Good |
| Holinka et al., 2018 | B | α | Bα | 2 | Very good |
| Iorio et al., 1999 | D | α | Dα | 4 | Fair |
| Kallala et al., 2015 | D | α | Dα | 4 | Fair |
| Kamath et al., 2015 | D | α | Dα | 4 | Fair |
| Kapadia et al. 2014 | B | δ | Bδ | 4 | Fair |
| Kasch et al., 2017 | B | α | Bα | 2 | Very good |
| Kurtz et al., 2008 | D | δ | Dδ | 5 | Poor |
| Kurtz et al., 2012 | D | γ | Dγ | 4 | Fair |
| Lavernia et al., 1995 | C | δ | Cδ | 4 | Fair |
| Lavernia et al., 2006 | D | γ | Dγ | 4 | Fair |
| Li Y. et al., 2013 | D | α | Dα | 4 | Fair |
| Musil et al., 2019 | D | α | Dα | 4 | Fair |
| Nichols et al., 2016a | D | α | Dα | 4 | Fair |
| Nichols et al., 2016b | B | α | Bα | 2 | Very good |
| Oduwole et al., 2010 | C | α | Cα | 3 | Good |
| Parvizi et al., 2010 | D | α | Dα | 4 | Fair |
| Puhto et al., 2019 | B | α | Bα | 2 | Very good |
| Reeves et al., 2018 | D | α | Dα | 4 | Fair |
| Ritter et al., 1996 | D | α | Dα | 4 | Fair |
| Sculco, 1995 | D | ε | Dε | 5 | Poor |
| Sousa et al., 2018 | B | α | Bα | 2 | Very good |
| Waddell et al., 2016 | D | α | Dα | 4 | Fair |
| Weber et al., 2018 | B | α | Bα | 2 | Very good |
| Yi et al., 2015 | D | α | Dα | 4 | Fair |
| Yao et al., 2020 | B | γ | Bγ | 3 | Good |
| Iqbal et al., 2020 | B | α | Bα | 2 | Very good |
| Rank guide: Adapted from Fukuda and Imanaka [17]; 1 {Aα}; 2 { Aβ; Bα; Bβ }; 3 { Bγ; Cα; Cβ; Cγ }; 4 { Bδ; Cδ; Dα; Dβ; Dγ }; 5 { Dδ; Dε } | | | | | |

Characteristics of excluded studies

| Authors, year | Cost year of study & currency | Cost estimate, 2019 USD; (CI); [SD] | Reason (s) for exclusion |
| --- | --- | --- | --- |
| Adeyemi et al, 2019 | 2013, USD | 14,010 [13,136] | Cost was a combination of reoperation and revision |
| Alp et al., 2016 | 2013, USD | 12,213 | Cost was a combination of RTHA and RTKA |
| Bozic et al., 2010 | 2006, USD | 30,785 (16,245 - 34,980) | Poor cost transparency |
| Burns et al., 2006 | 2005, USD | 11,373 | High risk of bias.  Cost was reimbursement |
| Clair et al., 2016 | 2013, USD | 43,007 (5,289 -115,700) | High risk of bias.  Cost was also a combination of gastrointestinal and pulmonary complications, reoperations, and revisions. |
| Dal-Paz et al., 2010 | 2008, USD | 3,219 (2,414 – 4,023) | Average cost was a combination of arthroscopic DAIR and revisions |
| Gow et al., 2016 | 2015, NZD | 29,308 | Cost was a combination of RTHA, RTKA and revision hip hemiarthroplasty |
| Holinka et al., 2018 | 2018, CZK | *Septic: 22,689*  *Aseptic: 14,526* | Cost was reimbursement |
| Kasch et al., 2017 | 2015, USD | 2-stage: *Septic: 15,674 Aseptic: 8,655* | Rebate prices |
| Kurtz et al., 2008 | 2006, USD | 35,098 | Poor cost transparency |
| Li Y. et al., 2013 | 2009, USD | 20,357.59 [ 8,477.91] | Cost was reimbursement |
| Nichols et al., 2016b | 2014, USD | 27,628.98 ± 23,436.63 | Cost was reimbursement |
| Puhto et al., 2019 | 2015, Euro | Septic: 28,998 (12,251 - 82,812) Aseptic: 21,028 (11,791 - 45,033) | Cost was a combination of RTHA and RTKA |
| Ritter et al., 1996 | 1993, USD | 3,288 | High risk of bias.  Cost was reimbursement |
| Sculco, 1995 | 1992, USD | 96,784 | High risk of bias.  poor cost transparency |
| Yi, H. et al., 2015 | 2010, USD | 128,592 | Cost was reimbursement |
| Yao et al., 2020 | 2018 USD | 2-Stage: 57912 (55177 - 62099) Aseptic: 25068 (24643 - 25475) | Hybrid costing (Healthcare provider + payer) |

CI: Confidence interval; SD: Standard deviation.

RTHA: Revision total hip arthroplasty; RTKA: Revision total knee arthroplasty.

CZK: Czech koruna; NZD: New Zealand dollar.
